# UK mothers' experiences of bottle refusal by their breastfed baby

**DOI:** 10.1111/mcn.13047

**Published:** 2020-06-17

**Authors:** Clare Maxwell, Kate M. Fleming, Valerie Fleming, Lorna Porcellato

**Affiliations:** ^1^ Faculty of Health, School of Nursing and Allied Health, Henry Cotton Building Liverpool John Moores University Liverpool UK; ^2^ Institute of Population Health Sciences, Department of Public Health and Policy, Whelan Building University of Liverpool Liverpool UK; ^3^ Public Health Institute, Exchange Station Liverpool John Moores University Liverpool UK

**Keywords:** breastfeeding, infant feeding, infant feeding behaviour, newborn feeding behaviours, quantitative methods, weaning

## Abstract

Little is known about bottle refusal by breastfed babies; however, an informal review of global online forums and social media suggested large numbers of mothers experiencing the scenario. This study aimed to explore UK mothers' experiences of bottle refusal by their breastfed baby in order to provide understanding of the scenario and enhance support for mothers experiencing it. A 22‐point online questionnaire was developed and completed by 841 UK mothers. Findings suggest that mothers introduced a bottle to their breastfed baby due to physical, psychological and socio‐cultural factors. Advice and support for mothers experiencing bottle refusal was not always helpful, and 27% of mothers reported bottle refusal as having a negative impact on their breastfeeding experience. When compared with eventual bottle acceptance, bottle refusal was significantly associated with previous experience of bottle refusal (*p* < .001), how frequently mothers intended to feed their baby by bottle and babies being younger at the first attempt to introduce a bottle (*p* < .001). This study provides a unique insight into the complexities of bottle refusal by breastfed babies and the impact it can have upon mothers' breastfeeding experiences. It generates knowledge and understanding that can help to inform practice and policies. In addition, a ‘normalising’ of the scenario could enable mothers, and those supporting them, to view and manage it more positively.

**Key messages**

- Reasons behind why mothers introduce a bottle to their breastfed baby are influenced by psychological, physical and socio‐cultural factors.
- Methods mothers use to manage bottle refusal can have limited success, and some babies will never accept a bottle.
- For some mothers, bottle refusal can have a negative impact on their breastfeeding experience.
- Bottle refusal by breastfed babies requires greater recognition and understanding in order for support to be enhanced for mothers experiencing it.
- A normalising of bottle refusal could counter the negative impact the scenario has for some mothers.

## 1 INTRODUCTION

Breastfeeding is clearly associated with short‐, medium‐ and long‐term benefits for mothers and infants (Victora et al., 2016). However, the United Kingdom has been described as a ‘bottle feeding culture’ (Dykes, 2006; Renfrew et al., 2007) and a ‘formula feeding nation’ (Brown, 2015). Such descriptions concur with figures from the last comprehensive UK Infant Feeding Survey (IFS) of 2010, which show that 80% of UK mothers have fed their baby with a bottle by 4–10 weeks of age (McAndrew, Thompson, Fellows, Speed, & Renfrew, 2012). Latest data for England show that only 32.8% of babies in England are totally (exclusively) breastfed at 6–8 weeks (PHE, 2020), with the 2010 IFS reporting less than 1% of UK mothers are exclusively breastfeeding their baby at 6 months of age (McAndrew et al., 2012). From this, it can be construed that the majority of UK babies are feeding by bottle rather than breast by around 6 weeks of age. For one group of mothers, however, circumstances are very different. They are breastfeeding, and when they wish to introduce a bottle to their baby, containing either expressed breast milk (EBM) or formula, their baby refuses to accept it.

Due to evidence that the introduction of a bottle to a breastfed baby can have a detrimental effect on breastfeeding duration (Forster et al., 2015; Isaia, Theodorou, Galanis, Nikolentzos, & Polyzos, 2017; O'Connor, Allen, Kelly, Gao, & Kildea, 2018), Step 9 of the World Health Organization (WHO, 2018) Ten steps to successful breastfeeding states: ‘Counsel mothers on the use and risks of feeding bottles, teats and pacifiers’. The UK Baby Friendly Initiative (BFI) standards, whilst making no explicit reference to the use of bottles and teats, include the need to ‘Support mothers to make informed decisions regarding the introduction of food or fluids other than breastmilk’ (UNICEF, 2012). In practice, mothers in the United Kingdom are advised to wait until breastfeeding is established in order to preserve breastfeeding and reduce any negative effect bottle introduction can have. However, breastfeeding mothers often want, or may need, to introduce a bottle to their baby (Gatrell, 2007; Johns, Forster, Amir, & McLachlan, 2013; McAndrew et al., 2012; McInnes, Hoddinott, Britten, Darwent, & Craig, 2013; Skafida, 2012), and when they are met with bottle refusal, anecdotal evidence suggests that this can incur negative consequences.

References to bottle refusal by breastfed babies in current literature are limited; however, online discussions within parenting forums, for example, babycentre, mumsnet and netmums, and Facebook, illustrate thousands of posts and threads in relation to the scenario. In addition, YouTube contains thousands of videos in relation to breastfed babies refusing a bottle, which, in turn, have elicited hundreds of thousands of online views (YouTube.com). This points to bottle refusal by breastfed babies being a potentially common scenario; however, the online references are circumstantial and remain unexplored; therefore, the context and background to the scenario is difficult to ascertain.

Our study has evolved from a significant gap in knowledge regarding bottle refusal by breastfed babies. The scenario has the potential to impact negatively upon breastfeeding and, as such, requires recognition and understanding to try to lessen this impact and to improve support for mothers experiencing it. We have therefore conducted an online questionnaire to explore the background and characteristics of bottle refusal, to capture demographic data of the mothers who experience it and to investigate potential relationships between bottle refusal and maternal demographics, timings and characteristics of the scenario.

## 2 METHODS

### 2.1 Defining bottle refusal

We developed a definition of bottle refusal following a review of the literature that revealed there was no prior agreed definition of the term. We undertook an informal scoping exercise with midwives at a Royal College of Midwives' conference and consulted online discussions between mothers. In order to provide as complete a picture of bottle refusal as possible, the definition included babies that had initially refused a bottle (and then possibly accepted) as well as those that were still refusing. In addition, it included both EBM and formula, in order to capture all scenarios surrounding bottle refusal. There was no minimum time in relation to when bottle refusal could occur; this was in order to capture those babies who potentially refused a bottle at birth. The following definition was created and embedded at the beginning of the questionnaire:

Bottle refusal is when a breastfed baby initially or continuously refuses to accept a bottle containing either expressed breastmilk or infant formula.

### 2.2 Questionnaire design

We designed a 22‐point online questionnaire capturing both quantitative and qualitative data (Table 1). We developed questions using the literature review and discussions on social media sites and forums. Demographic categories were derived from Office for National Statistics (ONS, 2011) classifications and the UK IFS 2010 (McAndrew et al., 2012). To maximise data collection, 21 out of the 22 questions were compulsory, and there was no option for partial completion of the questionnaire. Because of the exploratory nature of the questionnaire, mothers could select more than one option for some questions, and we included options for free text. We piloted the questionnaire with health professionals for face validity (*n* = 5) and mothers for content and readability (*n* = 10). Minor changes were made regarding wording to ensure greater clarity.

**TABLE 1:** Example questions

Question	Categories
1. Which baby did you experience bottle refusal with? If you have experienced it more than once, please complete regarding the most recent baby, if twins report on the oldest baby.	[1st] [2nd] [3rd] [4th] [other—please specify]
2. What is the sex of your baby?	[Male] [Female]
3. How long ago did you experience bottle refusal?	[up to 1 year ago] [up to 2 years ago] [up to 3 years ago] [up to 4 years ago] [up to 5 years ago] [experiencing it now]
4. At what age was your baby when you FIRST tried to introduce a bottle to it?	[free text]
5. Why did you want to introduce a bottle to your baby? (select all that apply)	[wanted to give up breastfeeding] [returning to work] [wanted some independence/social life] [wanted to spend time with other children] [other—please specify]
6. How often did you want your baby to feed from a bottle if it accepted one?	[every feed—no more breastfeeding] [daily—alongside breastfeeding] [occasionally not on a daily basis] [other—please specify]
7. Which method(s) did you use to try to introduce a bottle to your baby? (select all that apply)	[partner/family member/friend fed baby] [cold turkey—did not breastfeed baby until it accepted a bottle] [put expressed breast milk into a bottle] [tried different bottles/teats] [used a cup] [gave bottle when baby was not hungry] [gave bottle when baby was hungry] [other—please specify]
8. Which method(s) worked, that is, your baby accepted a bottle? (select all that apply)	[partner/family member/friend fed baby] [cold turkey—did not breastfeed baby until it accepted a bottle] [put expressed breast milk into a bottle] [tried different bottles/teats] [gave bottle when baby was not hungry] [gave bottle when baby was hungry] [nothing worked] [other—please specify]
9. Where did you go to for advice/support? (select all that apply)	[health visitor] [other mothers] [family and/or friends] [breastfeeding support groups] [internet] [did not seek advice] [other—please specify]
10. Which source(s) of advice/support were helpful to you? (select all that apply)	[health visitor] [other mothers] [family and/or friends] [breastfeeding support groups] [internet] [do not think any advice helped me] [not applicable as did not seek advice] [other—please specify]
11. How long OVERALL did it take for your baby to accept a bottle? That is, from your first attempt to the attempt that was successful. This could be in hours, days, weeks or months. If your baby is still refusing a bottle, please state this.	[free text]
12. What age was your baby when it accepted a bottle? If your baby is still refusing a bottle, please state this.	[free text]
13. Have you experienced bottle refusal previously?	[yes] [no]
14. Were you aware of bottle refusal by breastfed babies before this experience?	[yes] [no]
15. What impact did bottle refusal have upon your overall breastfeeding experience?	[positive] [negative] [had no impact] [other—please specify]
16. In hindsight is there anything you would have done to try to prevent bottle refusal occurring? (not compulsory)	[free text]

At three separate points in the questionnaire (Questions 4, 11 and 12), mothers were asked to report time and age‐related data in relation to their baby. This was in order to explore timings around bottle introduction and bottle acceptance (if it had occurred). In order to minimise ‘rounding’, we gave mothers the option to complete some of the time‐related questions in hours, days, weeks or months. Ages were converted to weeks using an age conversion calculator. To maintain data accuracy, and in response to possible maternal recall errors/bias, we developed the following calculation: age at introduction + length of time to acceptance = age at acceptance. Cases with a discrepancy of 2 weeks either way of the calculation result were excluded from the analysis for Questions 4, 11 and 12 (52 cases).

### 2.3 Inclusion criteria

The questionnaire aimed to recruit UK mothers who were experiencing, or who had experienced, bottle refusal by their breastfed baby. The following inclusion criteria were developed:

- UK mothers who have experienced bottle refusal by their breastfed baby in the past 5 years or who are experiencing it now;
- mothers whose baby was born after 37 weeks of gestation;
- mothers whose baby has no serious health problems;
- mothers >18 years; and
- mothers who could read and understand English.

A decision was made to include mothers who had experienced bottle refusal up to 5 years ago in order to increase recruitment and to capture mothers who had potentially experienced bottle refusal with more than one baby. The inclusion criteria were placed at the beginning of the questionnaire, embedded within the participant information form.

### 2.4 Recruitment

We sent the questionnaire URL link to five North West of England mothers who posted it on Facebook breastfeeding groups. We also sent the URL link to a participant of a mailing group for women from different ethnic backgrounds. This was a targeted attempt to reach mothers experiencing bottle refusal from ethnic minority groups. The URL to the questionnaire was open over a 2‐week period and was completed by 841 UK mothers.

### 2.5 Data coding and analysis

Responses from mothers who stated that their baby had not accepted a bottle were coded into the variable of ‘refusal’. Responses from mothers who gave an age at acceptance were coded into the variable ‘eventually accepted’. Preliminary descriptive analysis of data was undertaken using SPSS v.23. Frequencies were obtained for categorical variables, and descriptive statistics calculated for continuous variables. Further analysis was undertaken in relation to independent variables (maternal demographics, timings and characteristics of bottle refusal) and the key variable of ‘refusal/eventual acceptance’. Non‐parametric tests were used due to non‐normal distribution of data. Mann–Whitney *U* tests were undertaken to compare differences in continuous data and categorical variables. Kruskal–Wallis tests were undertaken to compare differences in continuous data and categorical variables with more than two categories. Spearman's Rank Order test (*rho*) was used to explore relationships between continuous variables. Chi‐square tests for independence were used to explore relationships between categorical variables; significant results were explored using standard residuals with significance determined by *z*‐scores >±1.96 or odds ratios (ORs) (Field, 2013). Significance for all two‐tailed probability tests was *p* < .005.

Qualitative data in the form of free text were exported directly into NVivo11 and analysed using a thematic analysis (Braun & Clark, 2013).

### 2.6 Ethical considerations

This study received full ethical approval from Liverpool John Moores University.

## 3 RESULTS

### 3.1 Participant demographics

A total of 841 UK mothers completed the online questionnaire. Only 39% of mothers reported eventual acceptance, with the majority (61%) reporting refusal at the time of completing the questionnaire. Over 95% of the mothers were white, >29 years in age and had left full time education at 19 years or over (Table 2). Although it was clear the questionnaire had ‘travelled’ UK wide, 40% of mothers resided in the North West. This can be attributed to the initial recruitment strategy. Ethnicity could not be analysed due to low numbers of mothers from ethnic minority groups. There were no significant associations between maternal demographics and refusal/eventual acceptance.

**TABLE 2:** Demographics and background/characteristics of bottle refusal

Demographic/background	*N* = 841, *n* (%)	Refusal *N* = 516 *n* (%)	Eventual acceptance *N* = 325 *n* (%)	Refusal/eventual acceptance *p*‐value
Age				.73
18–24	32 (3.8)	17 (3.3)	15 (4.6)	
25–29	158 (18.8)	93 (18.0)	65 (20.1)	
30–34	351 (41.7)	222 (43.0)	129 (39.9)	
35–39	239 (28.4)	149 (28.9)	90 (27.9)	
40+	60 (7.1)	35 (6.8)	24 (7.4)	
Missing value	1 (0.1)		2 (0.1)	
Ethnicity^a^				
White	806 (96.0)	496 (96.1)	310 (96.0)	
Mixed/multiple ethnic groups	20 (2.4)	11 (2.1)	9 (2.8)	
Asian/Asian British	9 (1.1)	6 (1.2)	2 (0.5)	
Black/African/Caribbean/Black British	5 (0.6)	2 (0.4)	2 (0.5)	
Other	1 (0.1)	1 (0.2)	0 (0)	
Missing value	1 (0.1)		2 (0.2)	
Age left full time education				.76
16 or under	29 (3.4)	17 (3.3)	12 (3.7)	
17	40 (4.7)	26 (5.0)	14 (4.3)	
18	93 (11.1)	53 (10.3)	40 (12.4)	
19 or over	678 (80.6)	420 (81.4)	257 (79.6)	
Missing value	1 (0.1)		2 (0.1)	
Employment status				.21
Employed	602 (71.6)	357 (69.5)	244 (75.1)	
Self‐employed	66 (7.8)	44 (8.9)	22 (6.8)	
Looking after family	119 (14.1)	82 (16.0)	37 (11.4)	
Student/unemployed	53 (6.3)	31 (6.1)	22 (6.8)	
Missing values	1 (0.1)	2 (0.1)		
Employment category^b^				
ONS categories 1–3^c^	492 (60.5)	296 (57.3)	196 (66.0)	
ONS categories 4–6^d^	125 (15.4)	79 (15.3)	46 (15.5)	
ONS categories 7–9^e^	23 (2.8)	14 (2.7)	9 (3.0)	
Missing values	173 (21.2)	127 (24.6)	46 (15.5)	
Sex (baby)			297	.79
Male	383 (45.0)	237 (45.9)	145 (44.8)	
Female	458 (55.0)	279 (54.1)	179 (55.2)	
Missing value			1 (0.1)	
Intended frequency to feed by bottle (if successful)				<.001
Every feed—no more breastfeeding	23 (2.7)	8 (1.6)	15 (4.6)	
Daily—alongside breastfeeding	184 (21.9)	102 (19.7)	82 (25.3)	
Occasionally—not on a daily basis/one off event	634 (75.4)	406 (78.7)	228 (70.1)	
Missing values	0 (0)			
Awareness of bottle refusal				.71
Yes	604 (71.8)	378 (73.2)	226 (69.7)	
No	236 (28.2)	138 (26.8)	98 (30.2)	
Missing values	1 (0.1)		1 (0.1)	
Previous experience of bottle refusal				.014
Yes	209 (24.6)	144 (27.9)	65 (20.1)	
No	631 (75.1)	372 (72.1)	258 (79.8)	
Missing values	1 (0.1)		2 (0.1)	
Impact on breastfeeding experience^f^				<.001
Negative	221 (27.5)	121 (24.5)	100 (32.5)	
Positive	58 (7.2)	46 (9.3)	12 (3.9)	
Mixture of negative and positive	109 (13.6)	64 (13.0)	45 (14.6)	
No impact	414 (51.6)	263 (53.2)	151 (49.0)	
Missing values	0 (0)			
Hindsight^g^				
Given a bottle earlier	303 (36.1)			
Considered giving a bottle earlier	90 (10.7)			
Would not have done anything differently	211 (25.1)			
Would not have given bottle in the first place	39 (4.6)			
Missing values	198 (23.5)			

^a^ Not analysed due to low numbers.

^b^ Twenty‐eight cases excluded, free text thematically analysed categories merged.

^c^ Managers, directors, senior officials, professional occupations, associate professional and technical.

^d^ Administrative and secretarial, skilled trades, caring, leisure and service.

^e^ Sales and customer service, process, plant and machine operatives, and elementary occupations.

^f^ Thirty‐nine cases excluded.

^g^ Non‐compulsory question free text thematically analysed.

### 3.2 Context surrounding bottle introduction

Mothers reported on the context surrounding their introduction of a bottle to their breastfed baby and included why, when and how often they intended to feed by bottle. Reasons to introduce a bottle to a breastfed baby were multifactorial (Table 3).‘Attending an event’ included undertaking exams, driving tests, weddings (including own) and funerals. Some mothers described challenging scenarios, ‘… my father had only weeks to live and was in intensive care and we needed her to take a bottle so I could spend some time with him’. ‘Other’ reasons included not wanting to feed in public and maternal illness, such as treatment for cancer, taking medication not compatible with breastfeeding and undergoing operative procedures, ‘I was facing an operation and wanted to ensure my baby could bottle feed beforehand …’.

**TABLE 3:** Reasons to introduce a bottle

Reason for bottle introduction	*N* = 841, *n* (%)^a^
Wanted partner/family to be able to feed baby	499 (59.3)
Wanted some independence/more social life	299 (35.6)
Wanted to spend some time with other children	129 (15.3)
Returning to work	121 (14.4)
Attending an event	39 (4.6)
Other	112 (13.3)
Wanted to give up breastfeeding	28 (3.3)

^a^ Mothers could select more than one option; therefore, total adds up to more than 100%.

The majority of mothers (75.4%) intended to feed their baby from a bottle occasionally/not on a daily basis or as a one off event if he or she accepted. Intended frequency to feed by bottle was significantly associated with refusal/eventual acceptance *p* ≤ .001, *r* = .174. Standard residuals revealed mothers who wanted to feed by bottle at every feed with no more breastfeeding reported significantly more cases of eventual acceptance (*z* = 2.7), *p* = .01 and significantly less cases of refusal (*z* = −2.2), *p* = .05. In addition, mothers who intended to feed their baby by bottle daily alongside breastfeeding reported significantly more cases of eventual acceptance (*z* = 2.3), *p* = .05.

Mothers reported the age of their baby when they had first attempted to introduce him or her to a bottle *Mdn* = 8 weeks (interquartile range [*IQR*] = 11, *min* = 0, *max* = 56, *R* = 56) and length of time to eventual acceptance *Mdn* = 9 weeks (*IQR* = 18, *min* = 0.1, *max* = 104, *R* = 103.9). Babies who eventually accepted were significantly older at first attempt to introduce a bottle than babies who refused (*Mdn* 12 vs. 8 weeks), *p* ≤ .001, *r* = .125. In addition, the older the baby was at first attempt to introduce a bottle was also significantly associated with a shorter length of time to eventual acceptance, *p* < .002.

### 3.3 Management of bottle refusal

Mothers reported on their management of bottle refusal, including methods used to overcome the scenario, sources of support used and, in hindsight, if there was anything they would have done to prevent bottle refusal. The majority of single methods used had a low success rate (<22%) (Table 4). Mothers also reported using a cup as a transition method, and some mothers reported ‘cup refusal’ alongside bottle refusal. Over half of mothers (59%) reported ‘nothing had worked’. ‘Other’ methods reported by mothers included sweetening the teat/milk, warming or cooling the milk and using ‘paced bottle feeding’.

**TABLE 4:** Comparison between methods used and methods that worked

Method	Method used *N* = 825, *n* (%)^a^	Method used that worked *N* = 825, *n* (%)^a^
Partner/family fed baby	791 (95.8)	167 (21.1)
Cold turkey	73 (8.8)	31 (42.4)
Used different bottles/teats	601 (72.8)	93 (15.4)
Used EBM in a bottle	777 (94.1)	100 (12.8)
Used a cup	359 (43.5)	69 (19.2)
Gave bottle only when baby was not hungry	282 (34.1)	16 (5.6)
Gave bottle only when baby was hungry	411 (49.8)	43 (10.4)
Tried different formula milks	180 (21.8)	15 (8.3)

Abbreviation: EBM, expressed breast milk.

^a^ Mothers could select more than one option; therefore, total adds up to more than 100%.

I tried everything … she wouldn't have a bottle or a cup and I felt totally trapped.

The majority of mothers (86%) sought various avenues of advice/support (Table 5). Of the mothers who did seek advice/support, 36% did not think it had helped them.

**TABLE 5:** Comparison between advice sought and advice that was helpful

Source of advice/support	Proportion of mothers who sought advice *N* = 720, *n* (%)^a^	Proportion of mothers who felt source of advice was helpful *N* = 720, *n* (%)^a^
Health visitor	324 (45.0)	55 (16.9)
Other mothers	446 (61.9)	197 (44.1)
Family/friends	385 (53.4)	108 (28.0)
Breastfeeding support groups	353 (49.0)	202 (57.2)
Internet	488 (67.7)	155 (31.7)

^a^ Mothers could select more than one option; therefore, total adds up to more than 100%.

Many health care professionals have just shrugged their shoulders in a way that suggested I just needed to get on with it. Some other breastfeeding mothers appeared appalled that I would want to give my baby a bottle in the first place and would ask ‘why on earth I might want an evening off?’ implicitly judging me for doing so.

All the comments I received from midwives and health visitors was that it was massively important to exclusively bf (breastfeed) and bottles were what bad mothers did. But then when I hit 6 months and he still wouldn't accept a bottle no one wanted to help and I felt trapped breastfeeding.

NHS staff were more concerned that baby would get nipple confusion and stop feeding. I was made to feel guilty for suggesting I needed the occasional night out or time with my husband and so wanted my baby to take a bottle as a result I stopped sooner.

Nearly half of mothers (46.8%) stated in hindsight they would have given a bottle earlier or considered giving a bottle earlier to prevent bottle refusal,

I would have offered a bottle within a week or so of birth and ignored advice about nipple confusion as now i (*sic*) am trapped breastfeeding and desperately want to stop. Really fed up and wish healthcare workers had been honest about this happening.

However, early introduction for some mothers made no impact on bottle refusal,

I tried early and regularly but it made no difference.

### 3.4 Impact

The impact of previous experience of bottle refusal on refusal/eventual acceptance and the impact of bottle refusal on breastfeeding experiences were explored (Table 2). A significant association between previous bottle experience and bottle refusal/eventual acceptance was found, *p* = .014, *r* = .088, with a calculated OR showing that the odds of bottle refusal were 1.53 times higher if a mother had experienced bottle refusal previously compared with if she had not experienced it (OR 1.53, 95% confidence interval [CI]: 1.01, 2.144), *p* = .005.

Over a quarter of mothers (27.5%) reported that bottle refusal had a negative impact upon their breastfeeding experience (Table 2). A significant association between impact of bottle refusal on breastfeeding experience was found *p* ≤ .001, *r* = .151, with more mothers reporting a negative impact with eventual acceptance (*z* = 2.1), *p =* .05 and less reporting a positive impact with eventual acceptance (*z* = −2.2), *p =* .05.

## 4 DISCUSSION

This study aimed to explore the background and characteristics of bottle refusal, to capture the demographic data of the mothers experiencing it and to investigate potential relationships between bottle refusal/eventual acceptance and maternal demographics, timings and characteristics of the scenario. It is the first of its kind to extensively explore mothers' experiences of bottle refusal by their breastfed baby, using a large‐scale sample and employing quantitative and qualitative methods.

The demographic profile of mothers within this study was similar to the demographics of breastfeeding mothers in the United Kingdom (GOV.SCOT.UK, 2018; McAndrew et al., 2012), although mothers from ethnic minorities, a further demographic associated with breastfeeding in the United Kingdom (McAndrew et al., 2012), were underrepresented in this research. We are unable, however, to propose a profile for those who typically experience bottle refusal as a non‐representative convenience sample was used.

All participants in this study had to have experienced, or be experiencing, bottle refusal. Some 61% reported that their baby was refusing a bottle at the time of completing the questionnaire. It is recognised, however, that some of the babies who were reported as refusing may go on to accept a bottle at a later date. In addition, it is also acknowledged that this study is not representative of all breastfeeding babies who are introduced to a bottle, as some will accept without any refusal.

Reasons why mothers wished to introduce a bottle to their breastfed baby exhibited social, physical, economic, cultural and environmental influences, with such influences being found previously to contribute to the dynamics of breastfeeding (Hoddinott, Seyara, & Marais, 2011; Radzyminski, 2016; Rollins et al., 2016). The demands of breastfeeding appeared to compete with the demands and needs of mothers' everyday lives in some cases. This has been voiced by mothers previously (Emmot, Page, & Myers, 2020; Hoddinott, Craig, Britten, & McInnes, 2012; Lavender, McFadden, & Baker, 2006; Spencer, Greatrex‐White, & Fraser, 2014) and has been found to underpin mothers' decisions to formula feed (Andrew & Harvey, 2011; Crossland et al., 2016; Lee & Furedi, 2005; Ryan, Team, & Alexander, 2013).

For some mothers, the decision to introduce a bottle may not be entirely their own, due to them facing hospitalisation or being unwell. Such circumstances are comparable with the ‘life events’ described in Hauck and Irurita's (2003) study, where impromptu weaning from the breast was required. It also echoes findings from McInnes et al. (2013), where tangible reasons for the introduction of formula, such as illness or separation, were not always within maternal or parental control and a ‘crisis bottle’ was required (McInnes et al., 2013, p. 9). Maternal illness has the potential to be further complicated when bottle refusal occurs, which is important to note given the event of the Covid‐19 pandemic.

This study found that mothers who intended to feed their baby by bottle more frequently reported more cases of eventual acceptance. This association may be influenced by mothers being more determined in their efforts for their baby to feed from a bottle, in particular those who wished to discontinue breastfeeding. This theory is supported by Hauck and Irurita (2003) who found that once mothers had made the decision to wean their baby from the breast, they persevered even when faced with their baby's opposition. Studies previously describe features of maternal character/personality including determination, perseverance and self‐efficacy as factors in overcoming breastfeeding challenges and increasing breastfeeding duration (Brown, 2014; Burns, Schmied, Sheehan, & Fenwick, 2010; Hegney, Fallon, & O'Brien, 2008; Jardine, McLellan, & Dombrowski, 2017; Ricotti, Apekey, & Gatenby, 2015; Williamson, Leeming, Lyttle, & Johnson, 2012). From this, it could be construed that maternal determination being an implicit factor in eventual acceptance is a plausible one. It could also be hypothesised that these mothers tried more regularly with a bottle and followed a routine in order to achieve acceptance. This is supported by studies that have found associations between feeding patterns and infant feeding outcomes (Caton et al., 2014; Hittner & Myles, 2011; Neighbors, Gillespie, Schwartz, & Foxman, 2003; Nekitsing et al., 2016; Shim, Kim, & Mathai, 2011). However, mothers also reported ‘trying everything’ and ‘early and regularly’ and still being met with refusal. Furthermore, data were not collected on how often mothers tried their baby with a bottle, so these are suppositions only.

Few studies focus on how mothers wean their baby from the breast and those that have depicted it as a potentially difficult and, at times, lengthy process (Eccleson, 2005; Egan, 1988; Hauck & Irurita, 2003; Neighbors et al., 2003; Williams & Morse, 1989). Methods used to manage weaning off the breast are purely anecdotal (Egan, 1988) and, as shown in this study, often unsuccessful. Studies have shown significant differences between the mechanisms of breastfeeding and bottle feeding (Aizawa, Mizuno, & Tamura, 2010; França, Sousa, Aragão, & Costa, 2014; Sakalidis & Geddes, 2015), with breastfeeding associated with a wide‐open mouth and bottle feeding with a pursed mouth (Woolridge, 1986). In addition, breastfeeding creates a ‘vacuum’ action, with bottle feeding undergoing a ‘compression’ action (Geddes & Sakalidis, 2016). Bottle refusal could therefore be due to a baby's ‘limited ability to adapt to various oral configurations’ (Neifert, Lawrence, & Seacat, 1995, p. 126). In response to this, there is a commercial emphasis in the United Kingdom on bottle refusal being ‘solved’ by bottles and teats that manufacturers aim (and claim) to emulate the mechanism of breastfeeding, prevent nipple confusion and alleviate bottle refusal (mimijumi.com, minibe.co.uk and tommeetippee.co.uk). However, early work by Sameroff (1968) and Wolff (1968) and more recently by Moral et al. (2010) has found that babies are able to adapt between the differing sucking mechanisms and it is also evident that some breastfed babies accept a bottle straight away with no refusal. Thus, to isolate bottle refusal as being due to differences in physiological retrieval of milk alone would be somewhat presumptuous and dismisses the non‐nutritional benefits of breastfeeding (Entwistle, 2014; Gibbs, Forste, & Lybbert, 2018; Gribble, 2006; Gribble, 2009; Papp, 2014; Weaver, Scoefiled, & Papp, 2018), which are likely to contribute to refusal in some cases.

The majority of mothers in this study believed ‘early introduction’ of a bottle was key to preventing bottle refusal. Interestingly, however, this belief is contradicted, as babies who were more likely to eventually accept a bottle were older when first introduced to one—although this finding should be viewed with caution as babies who accept a bottle immediately are not included in the study sample. When to introduce a bottle to a breastfed baby is complex. There is the potential for ‘nipple confusion’, whereby a breastfeeding baby who is introduced to a bottle makes a preference for bottle feeding to the detriment of breastfeeding (Neifert et al., 1995). However, the causal link between bottle feeding and nipple confusion is yet to be proven (Zimmerman & Thompson, 2015). In addition, current evidence indicates that bottle introduction can result in a negative effect on breastfeeding duration (Forster et al., 2015; Isaia et al., 2017; O'Connor et al., 2018), although this is mainly in relation to formula rather than EBM. No studies are able to give an optimum time for introduction of a bottle, and the current anecdotal advice to wait until breastfeeding is established is problematic in that there is no definition of the term ‘established’, which is likely to be individualised. However, there appears to be a strong belief held by mothers in this study that early bottle introduction can preclude bottle refusal, which could be detrimental to breastfeeding duration.

Advising mothers on bottle refusal can be challenging for health professionals. Apart from there being no evidence to draw upon to underpin support, there is the potential ‘dilemma’ of health professionals being seen to support mothers to formula feed, which conflicts with the benefits of breastfeeding (Trickey & Newburn, 2014) and as discussed earlier can be detrimental to the duration of breastfeeding. Furthermore, the issue of nipple confusion remains at the forefront of some health professionals' advice in this study even though the evidence surrounding it is inconclusive. The use of cold turkey is of concern, given that it can lead to dehydration in the baby (Staub & Wilkins, 2012) and mastitis and/or breast abscess in the mother due to acute cessation of breastfeeding (Noonan, 2010). For mothers experiencing bottle refusal, recognition of the scenario, and support for mothers to ‘work around’ it, is needed. Information regarding the potential risks of cold turkey is required for mothers who employ this as a method. In addition, alternative feeding options to breast, bottle and cup warrant further exploration, with finger feeding, syringe feeding, straw, paladai and spoon feeding being potential, effective substitutes when bottle refusal occurs.

Interestingly, mothers in this study who had experienced bottle previously were more likely to report refusal rather than eventual acceptance. This could be explained by these mothers being more realistic in their knowledge that acceptance was not always readily achieved. They may have been better prepared for accepting continued refusal and less likely to pursue acceptance. Furthermore, they could be replicating their previous (unsuccessful) management of bottle refusal, particular in terms of bottle feeding per se that can be dismissed as a skill that both mother and baby need to learn. This is likely to be exacerbated by health professionals being found to prioritise breastfeeding whilst limiting information surrounding bottle feeding during infant feeding discussions (Crossley, 2009; Lagan, Symon, Dalzell, & Whitford, 2014; Lee & Furedi, 2005; Leurer & Misskey, 2015).

Bottle refusal by a breastfed baby means exclusive breastfeeding has the potential to continue for longer, which, from a physical health perspective, is a positive outcome (Victora et al., 2016). However, the lens through which this is viewed is not always a positive one, and the increased duration of breastfeeding should be balanced against the potential negative, psychological impact bottle refusal may generate. Mothers in this study whose baby did accept a bottle were more likely to report a negative impact than those who refused. This indicates that the impact of bottle refusal upon breastfeeding experience is not solely outcome driven: an important finding for those supporting mothers experiencing the scenario. A ‘normalising’ of bottle refusal as a natural response by a healthy, breastfed baby could alleviate some of the negativity surrounding the scenario. For this to happen however, it is acknowledged a socio‐cultural shift would be required, ‘normalising’ breastfeeding and reversing the UK bottle feeding culture (Brown, 2015; Leahy‐Warren et al., 2017).

### 4.1 Strengths and limitations

This study is not without its limitations. Maternal recall was up to 5 years, which could have affected the accuracy of mothers' answers. Although checks for accuracy were employed in relation to mothers' responses to ‘time’ and ‘age‐related’ data. It is clear, due to cases that had to be excluded that recall was not always accurate. The nature of the online convenience sample would limit the application of the findings to the wider population due to self‐selection bias, particularly from mothers who had a negative experience and wished to present this. In addition, the sample was underrepresented by mothers from ethnic minority groups: the mothers most likely to breastfeed in the United Kingdom (McAndrew et al., 2012). The strengths of this study lie in the sample size of 841 mothers, which provides a unique and valuable insight into a large number of UK mothers' experiences of bottle refusal by their breastfed baby.

## 5 CONCLUSION

This study has illustrated UK mothers' experiences of bottle refusal by breastfed babies. It provides a rationale for recognition and understanding of bottle refusal in order to enhance support and advice for the mothers experiencing it. A ‘normalising’ of bottle refusal by breastfed babies, framing it as a natural response by a healthy, well baby, is needed in order to help counter the negative impact the scenario has for some mothers. In addition, a focus on supporting mothers to breastfeed alongside bottle refusal has the potential to encourage mothers to continue to breastfeed exclusively for longer. Importantly, the exploration of other feeding receptacles to be used temporarily when mothers are unwell or facing separation from their baby is warranted: a recommendation that is particularly pertinent given the event of the Covid‐19 pandemic.

## CONFLICTS OF INTEREST

The authors declare that they have no conflicts of interest.

## CONTRIBUTIONS

CM performed the research. CM, KMF, VF and LP designed the research study. CM and KF analysed the data. CM, KMF, VF and LP wrote the paper.
